# Why did doctrinal religions first appear in the Northern Subtropical Zone?

**DOI:** 10.1017/ehs.2023.13

**Published:** 2023-05-17

**Authors:** R.I.M. Dunbar

**Affiliations:** Department of Experimental Psychology, University of Oxford, Radcliffe Observatory Quarter, Oxford OX2 6GG, UK

**Keywords:** Holocene, scalar stress, pathogen load, latitude, language evolution

## Abstract

Doctrinal religions that involve recognised gods, more formal theologies, moral codes, dedicated religious spaces and professional priesthoods emerged in two phases during the Neolithic. Almost all of these appeared in a narrow latitudinal band (the northern Subtropical Zone). I suggest that these developments were the result of a need to facilitate community bonding in response to scalar stresses that developed as community sizes increased dramatically beyond those typical of hunter–gatherer societies. Conditions for population growth (as indexed by rainfall patterns and the difference between pathogen load and the length of the growing season) were uniquely optimised in this zone, creating an environment of ecological release in which populations could grow unusually rapidly. The relationship between latitude, religion and language in contemporary societies suggests that the peculiar characteristics of the northern (but not the southern) Subtropical Zone were especially favourable for the evolution of large scale religions as a way of enforcing community cohesion.

## Graphical abstract

**Figure:**
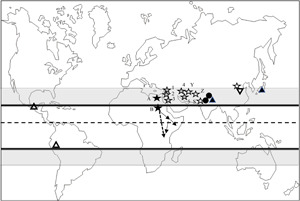

**Social media summary:** Doctrinal religions first appeared in the Levant because of the unique conditions favouring population growth.

There is a long-established consensus that, broadly speaking, religions evolved in at least two major phases: an early informal animist phase (still widely represented among contemporary hunter–gatherer societies), followed by a phase of more formally organised doctrinal religions (associated with a more sedentary lifestyle) (Peoples et al., 2016; Dunbar, 2022a). Hunter–gatherer religions typically have no specific gods or priestly caste, do not distinguish between the physical and the spiritual realms, are ‘animist’ in character (recognise a wide range of spirits or ‘forces’ associated with environmental features), rarely have divinely (as opposed to socially) justified moral codes or formal rituals, and are normally immersive (i.e. based on trance states). In contrast, doctrinal religions are characterised by belief in distinct gods (many of whom may be ancestors) that occupy a transcendental realm, have prescribed forms of ritual, formal religious spaces such as temples and a specialist priesthood. The earliest archaeological evidence for doctrinal religions is associated with settlements of the Neolithic from about the tenth millennium BP onwards (Hodder, 2010; Dietrich et al., 2012). Although these doctrinal religions are typically polytheistic, many have a ‘Supreme (Creator) God’ along with a panoply of lesser gods, ogres and deified ancestors (many of whom directly influence human activities).

Between the third and second millennia BP, some of these doctrinal religions give rise to a second phase of more institutionalised forms of doctrinal religions – the ‘revealed’ religions that form the basis for most of the contemporary world religions. These phase-2 doctrinal religions differ from the earlier phase-1 doctrinal religions in that they are typically associated with a specific named founder (who ‘revealed’ the religion to the world) and have a divinely justified moral code associated with a ‘Moralising High God’ who typically takes an active moral interest in human behaviour (as opposed to typical phase-1 gods who usually just demand propitiation via sacrifices). In contrast to the phase-1 doctrinal religions (which were, and still are, typically highly localised on a tribal scale), most of these phase-2 doctrinal religions grew very rapidly and, over the course of just a few centuries, transcended local tribal boundaries to become ‘world religions’ that numbered their adherents in the millions rather than the thousands. Many of these are monotheistic, although in some cases (such as Jainism and Buddhism) this may take the form of a more indistinct ‘universal principal/power’ (in the latter cases, at least, associated with the concept of *karma*). These phase-2 doctrinal religions are sometimes referred to as Axial Age religions.

Lightner et al. (2022) provide useful (albeit indirect) evidence that belief in formal gods (their ‘moralising gods’ category, equivalent to my phase-1 doctrinal religions) are rare in hunter–gatherer societies, but common in post-settlement societies, and that phase-2 religions (equivalent to their ‘moralising high gods’ category) appear only in more complex societies. They use ‘jurisdictional hierarchy level’ (number of societal administrative levels) as a definition of societal complexity, which, although not necessarily correlated with actual living-group size, is probably an adequate proxy for present purposes. Similarly, in the limited context of Austronesian societies (all of which are village-living), Sheehan et al. (2023) have shown that religious specialists (priests) are widely characteristic of societies that have cultivator, herding and/or fishing economies. Doctrinal-type religions emerged in many parts of the world as local populations began to become more spatially concentrated in the aftermath of adopting agriculture. In most of these cases, however, we have no idea when this actually happened other than that it postdated the local adoption of agriculture (in effect, any time between about the fifth millennium BP and the present).

These phases in the evolutionary history of religion should not be seen as typologically distinct (in fact, they shade into each other), or as some kind of inevitable evolutionary process. Rather, they are properly interpreted as a continuum in which novel elements are successively added without removing the components of the preceding phase. The world religions share many features in common with immersive hunter–gatherer trance-based religions (including trance and visions), and with phase-1 doctrinal religions (e.g. worship of saints/ancestors, forecasting, healing, belief in the capacity of the spirit world to intervene in the physical world: Dunbar 2022a). These phases differ mainly in scale and organisational complexity, and are best understood as structural responses to the need to manage increasingly large, concentrated populations. For present purposes, however, we can regard the three phases as being distinct enough to ask why they originally appeared where and when they did.

There have been a number of recent attempts to understand the origins of the doctrinal (phase-1) religions. Botero et al. (2014) examined the correlates of belief in theologically endorsed moral codes in nearly 600 ethnographic societies and found that such beliefs were more likely where the society is complex and structurally multilevel, resource abundance is low, the climate unstable and animal husbandry more common. In a phylogenetically controlled analysis of contemporary hunter–gatherer societies, Peoples and Marlowe (2012) found that Moralising High Gods emerge mainly as a late-phase development only in pastoralist economies (see also Peoples et al., 2016). In another phylogenetically controlled study of hunter–gatherer societies, Watts et al. (2022) found that the crucial prerequisite for the appearance of a specialised priestly class (a key marker of doctrinal religions) is food storage – probably because food storage is necessary to ensure sufficient surplus to support a class of professional priests who do not produce their own food.

Other studies have focussed on the origins of the phase-2 (Axial Age) doctrinal religions, not least because these are easier to identify. Baumard et al. (2015), for example, collated historical data on a number of economic, social and demographic measures across the three millennia up to 2000 BP in the three regions that generated most of the Axial Age religions (the Yellow/Yangste River basin in eastern China, the Ganges plain in northern India and the eastern Mediterranean). The variable that changed most was per capita energy production (based on estimated agricultural output), with population density as the next best (and probably more underlying) predictor. Whitehouse et al. (2023) collated data from some 300 historical societies covering the last 10,000 years and mapped the changes in social complexity either side of the appearance of formal moral codes and a belief in ‘moralising supernatural punishment’ (typically associated with High Gods, both being markers of Axial Age religions). They found that socio-political complexity (indexed by a somewhat idiosyncratic mix of traits that included population size, hierarchical social structures, legal codes, judiciaries, infrastructure such as canals and roads, and the possession of calendars, writing and metal coinage) characteristically increases 500–1000 years before the appearance of moralising supernatural punishment and was invariably associated with polity sizes in excess of a million people.

Taken together, these findings suggest that community size may have been a precipitating factor in the rise of doctrinal religions at both transitions. If so, then this suggests that the underlying context may have been the difficulty of maintaining social cohesion and community integrity as living-group size increased through time. Scalar stresses of this kind have been widely documented in human societies ever since they were first proposed by Johnson (1982). In contemporary small scale societies, living in close spatial proximity is stressful and leads to significant levels of dysfunctionality in at least two different respects. One is that crowding adversely affects female fertility in mammals in general, including humans (Dunbar & Shultz, 2021a); the other is that relationships between individuals become increasingly fractious as living-group size increases.

Just how fractious relationships are in these contexts is made clear by two sets of data. One is the evidence that homicide rates increase linearly with living-group size in hunter–gatherer societies (Dunbar, 2022b). Extrapolating the regression for these data suggests that all mortality would be due to homicide once living-group size exceeded ~90 people (of all ages). Since homicide tends to afflict younger rather than older individuals (and, in small-scale societies, often differentially affects women: Pardoe, 2016), this effectively sets an upper limit on the number of people that can live together in the absence of any social mechanisms to manage disputes. Hunter–gatherers solve this problem by dispersing their communities (clans, which typically number 100–200 people) into a number of smaller camp groups (bands: typical size 30–50) so as to maintain living-group size below the threshold that would make communal life intolerable (Dunbar, 2022b). The second piece of evidence comes directly from the Holocene Levant: the structural integrity of living sites (indexed by the degree of similarity in the material culture of different parts of the site) declined dramatically through time as settlements became larger (Coward & Dunbar, 2014: fig. 17.1b). In other words, as the Holocene progressed, settlements became increasingly socially fragmented as they got bigger, creating more opportunities for partisan disputes and differentiation.

To be able to live in permanent settlements significantly larger than the band sizes observed in contemporary hunter–gatherers required the adoption of social institutions that allowed conflict within the group to be managed (Dunbar, 2022b). In the small scale village-sized societies of 100–300 people typical of contemporary horticulturalists (the type of society that would have characterised the early phase of sedentism in the Holocene), the kinds of social institutions adopted relate mainly to personal relationships and, in particular, the control of volatile male behaviour: they include (non-hereditary) charismatic leaders, men's clubs, marital arrangements and obligations, within- and between-community feasts, and communal singing and dancing (Dunbar, 2022b). Feasting, singing and dancing are all known to up-regulate both the endorphin system the principal neuropharmacological mechanism that underpins social bonding in all primates and humans: Dunbar 2010, 2021; Machin & Dunbar 2011; Pearce et al. 2017, 2018) and the sense of communal bonding.

That religion might play a role in community bonding in larger scale societies was originally suggested by Carneiro (1967) and Adler and Wilshusen (1990). Their respective analyses of ethnographic data suggested that more formal (i.e. phase-1 doctrinal) religions begin to emerge when community sizes reached a threshold somewhere in the region of ~400 individuals. Direct support for the suggestion that religion allows larger community sizes to remain stable for longer is provided by historical evidence from nineteenth-century American utopian communes: religious foundations were larger at foundation (~150 vs. ~50) and survived longer (by an order of magnitude) than strictly secular ones (Dunbar & Sosis, 2018). A religious framework seems to allow a community to manage more effectively the stresses that inevitably arise when living in close spatial proximity. One reason for this is that the rituals of religion upregulate the brain's endorphin system, thereby creating a sense of belonging, commitment and tolerance (Dunbar, 2020a, 2021; Charles et al., 2020, 2021).

The fact that religion is a universal characteristic of humans inevitably raises the functional question of what fitness benefit it provides. While it has been suggested that religion might be adaptive for a variety of reasons (as a framework for explaining and controlling the world, as a palliative for coping with life's exigencies, to manage sickness), I argue that, notwithstanding these secondary benefits, the principal reason religions evolved was as part of the suite of community bonding mechanisms (Dunbar, 2008, 2013, 2020a, 2022a; Coward & Dunbar, 2014). It is on this *social* aspect of the adaptive value of religion that I want to focus here.

Note that my concern is not with the kinds of social organisation that characterise particular tribal societies but simply with the *demographic* context and the social and physiological stresses that this imposes on the ability to maintain stable social groupings of any significant size. Zhou et al. (2005), Hamilton et al. (2007), Layton & O'Hara (2010), Lehmann et al. (2014) and Bird et al. (2019) have shown that hunter–gatherers live in hierarchically nested societies, with communities (or clans) of ~150 dispersed in living groups (or bands) of 30–50 distributed around the community's ranging area, while these communities in turn cluster into successively higher level groupings (mega-bands and tribes). These layers of society were already present in hunter–gatherer societies long before the Holocene – indeed, they can be tracked in trading networks in Late Palaeolithic communities (Pearce 2014; Pearce & Moutsiou 2014). The sizes of these layers are fractally related (Dunbar, 2020b), with the specific sizes of the different layers functioning as attractors where information flow round the network is optimised (West et al., 2020, 2023; Dunbar & Shultz, 2021b). These structures did not change during the transition to permanent settlements: they still define contemporary social networks (Dunbar, 2020b) as well as the organisational structure of early state societies (Narrol, 1956; Kosse, 1990; Sandeford, 2018; Hamilton et al., 2020). They do, however, provide a natural basis whereby progressively larger settlements can be built up by including successive layers of people who are familiar and share common interests.

To make the case for a role for doctrinal religions in this process, I first show that almost all the major large-scale contemporary Axial Age religions arose in the northern Subtropical latitudinal zone and that, during the early Holocene, this zone was characterised by rapid population growth. I will suggest that this was due to the unique convergence of two opposing environmental factors (the latitudinal distributions of pathogen load and the length of the growing season) that directly influence population growth, combined with high levels of rainfall. My claim is that doctrinal religions arose in response to the scalar stresses that were a direct result of rapidly increasing community size and the constraints that this would have placed on the capacity to live in settlements of any size: large-scale settlements would have been impossible without the development of doctrinal-type religions. I then suggest that we can gain further insights into this from the contemporary latitudinal distribution of religions and languages.

## Methods

Since phase-1 doctrinal religions pre-date writing and their origins are inevitably difficult to identify archaeologically, I use the geographical distribution of the point of origin of the phase-2 Axial Age religions as a more reliable guide to where at least these doctrinal religions first developed. Historically, they date from a period that is only about five millennia after the earliest evidence for doctrinal religions, making them an historically reasonable proxy. Whitehouse et al. (2023) list a number of religions identified by beliefs in MHG (Moralising High Gods). I excluded any that were secondary (e.g. Judaism in Yemen) and any phase-1 doctrinal religions (e.g. the Akan). I supplemented this by a literature search for ethnographic monotheistic religions associated with contemporary tribal groupings. These yielded only a very small number of additional cases (Atenism in XVIIIth Dynasty Egypt; Shangdi and Mohism in Shang Dynasty China; and the Cushitic and Nilotic tribes of eastern Africa). I have included Jainism and Buddhism on the grounds that, although neither recognises a formal God, both conceptualise a form of ‘Universal Force’ that regulates the human universe. On these grounds, one might legitimately include Confucianism and Daoism (both of which date from the third millennium BP) since, although they do not recognise gods as such, they articulate formal moral codes that derive from a higher principle (*Tian*, or the rather mystical ‘Gateway to Heaven’ in the case of Confucianism; the *Dao*, or ultimate principle underlying the universe, in the case of Taoism) within which those who have lived a ‘good life’ become united or immersed. This has led to both being considered as meaningful religions, but also as social philosophies.

The monotheistic Cushitic tribes originated in the central Nile Valley, perhaps in the vicinity of Khartoum (latitude 15° N), some time around 7000 BP, and moved into highland Ethiopia and the deserts to the south and east over the following millennia. Although many of the tribes converted to Coptic Christianity or Islam during the first millennium in the Horn of Africa, those who migrated further south (e.g. the Oromo, El Molo and Rendille) continue to worship the ancestral Cushitic sky-god *Waaq* (believed to derive from the pre-Abrahamic religion of Nubia; Blench, 1999; Hassen, 2015; Shriner et al., 2016). The Nilo-Hamitic tribes also derived from the Central Nile Valley, albeit sometime after the Cushitic tribes, and migrated down into East Africa at various stages between the third and first millennium BP. Like the Cushitic tribes, they also worship a singular sky-god (variously named *Engai* by the Maasai, *Nyasaye* by the Luo, *Asis* by the Kalenjin). However, in addition, some also sacrifice to spirits (usually ancestors) that are able to intervene in human affairs.

There are a number of large-scale centralised state religions (Shinto in Japan, and the religions of the Aztec and Tiwanaku/Inca empires in Central and South America) that I include even though they have strong animist elements and lack a Moralising High God (Kolata, 1993; Brumfiel, 2001; Janusek, 2006) on grounds of the size of the population to which they applied (or, in the case of Shinto, still apply); they may represent a transitional state between the two phases of doctrinal religion.

I considered the Akan, Igbo and Tikar in West Africa and the Herero/Himba in southwest Africa as possible candidates because these have sometimes been described as being monotheistic; however, I concluded that their respective religions were too similar to the religions of other Bantu and Bantoid tribal groups: all recognise a Supreme, or Creator, God (who generally does not take a lot of interest in human affairs, unless they fail to perform the required rituals and sacrifices) plus a pantheon various minor gods, ogres and/or ancestors (to whom petitions and rituals for wellbeing are mainly directed, and who both bestow the moral code and punish defaulters) (Werner, 1933; Egboh, 1972; Pobee, 1976; Afigbo, 1980; Klein, 1996; Bollig, 2009; McCulloch et al., 2017). I concluded that, despite the scale at which some of these operate, they are all better described as forms of ‘ancestor worship’ (Pobee, 1976), and hence as *bona fide* phase-1 doctrinal religions.

Data on contemporary growing season length as a function of latitude are from Håkonson and Boulion (2001) and those for contemporary disease prevalence as a function of latitude are from Bonds et al. (2012). Data on the number of religions and disease load by country are from Fincher and Thornhill (2008) and Fincher et al. (2008), and on the geographic distribution of languages from Nettle (1999). For simplicity, a country's latitude is taken to be that of its capital city. The data are given in the Supplementary Information *Datafile*.

## Results

### Origin of the Axial Age religions

**Figure 1.**
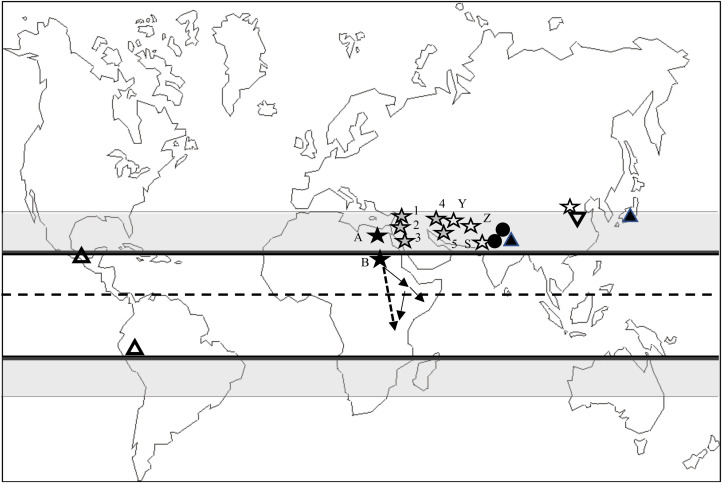
Place of origin of the main phase-2 doctrinal religions. Dashed line is the Equator. The solid lines either side demarcate the Tropics, and the grey-shaded areas are the subtropical zones. 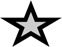 Place of origin of the Abrahamic religions: 1, Christianity (c. 30 AD); 2, Judaism (c. 2500 BP); 3, Islam (c. 600 AD); 4, Mandaeanism (c. 100 AD); 5, Manicheism (c. 250 AD). Druze (c. 1000 AD) and Bahai (c. 1850 AD) faiths are considered derivatives of Islam. 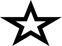 Other monotheistic religions in Asia (left to right): Yazidis (Y) (c. 1150 AD), Zoroastrianism (Z) (fourth millennium BP), Sikhism (S) (c. 1500 AD) and, in Shang Dynasty China, Shangdi (fourth millennium BP) and its derivative Mohism (c.2400 BP). 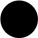 Non-theistic world religions with belief in a ‘universal force (principle)’: Jainism (c. 3000 BP) and Buddhism (c. 2500 BP). 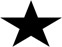 Monotheistic tribal religions: (a) Atenism (XVIIIth Dynasty, Pharaonic Egypt, fourth century BP); and (b) two groups of largely pastoralist tribes that have their origin in the central Nile valley – the Cushitic tribes (including the Oromo, Somali, Boran, Rendille and others) now inhabiting central Ethiopia, Somalia and northern Kenya (solid lines), and the Nilotic tribes (including the Anuak, Shilluk, Acholi, Luo, Samburu, Maasai and others) now mainly inhabiting southern Sudan and western East Africa (dashed line). 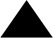 Major state polytheistic religions: Hinduism (northern India) and Shinto (Japan). 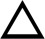 Polytheistic state religions in the New World: Maya (250–1700 AD) and Aztec (1325–1520 AD) empires (southern Mexico); Tiwanaku (Bolivia; 600–1000 AD) and Inca (Peru/Bolivia; 1200–1530 AD) empires. 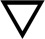 Confucianism and Daoism, third millennium BP China.

Figure 1plots the place of origin of all the major phase-2 (Axial Age) doctrinal religions, as well as the various ‘state’ religions and the monotheistic tribal religions. The distribution in Figure 1 is clearly not random. With the exception of the Tiwanaku/Inca cultural complex in Bolivia/Peru, all of these are associated with the northern Subtropical Zone. At the time they were founded, all were associated either with agricultural economies or with pastoralism. Not only did all the Axial Age religions originate within or adjacent to the northern Subtropical Zone, but, with just one exception, so did both the various state religions and the ethnographic monotheistic religions. It is worth noting that the Akan, Igbo and Tikar (whose religions I considered as possible candidate phase-2 doctrinal religions) are all thought to have originated from much further north than their current locations in the West African forest belt (Afigbo, 1980; Klein, 1996; McCulloch et al., 2017), most likely in the Sudanian grassland belt or the Sahel (the latter would have placed them within the northern Subtropical Zone).

### Holocene climate of the northern Subtropical Zone

From 15,000 BP until around 5000 BP (the African Humid Period), the northern Subtropical Zone was much more luxuriant than it is now, a phenomenon known as the ‘Green Sahara’ (Le Houérou, 1997), providing unusually benign climatic conditions right across Africa, the Middle East and Asia. Its defining characteristic was a rapid increase in ground water over a period of just a few decades owing to a northward shift in the summer monsoon (Kuper & Kröpelin, 2006). During this period, the Sahara was a rich, well-watered environment that contained several very large mega-lakes as well as a large number of substantial permanent rivers comparable in size with the modern Nile (Nicholson & Flohn, 1980; Street & Grove, 1976; Drake & Bristow, 2006; Drake et al., 2011; Revel et al., 2014; Lécuyer et al., 2016; Kaniewski et al., 2018; Lüning & Vahrenholt, 2019). During this period, the Nile had much higher water levels than it does now (Kaniewski et al., 2018). These lacustrine and riverine environments supported abundant fish, hippos and crocodiles, and a rich variety of terrestrial mammals (including baboons, rhinos, warthogs and gazelles) that are no longer found there (van Neer et al., 2020). The northern limit for the distribution of baboons (genus *Papio*), for example, is now 1400 km to the south of where it was at the peak of the African Humid Period.

**Figure 2.**
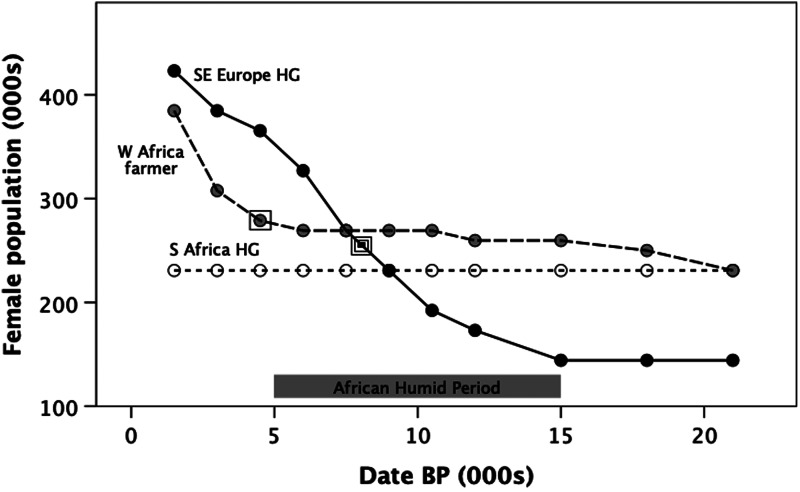
Estimated total female population size during the Holocene, from a genetic coalescent-based analysis applied to mtDNA haplogroups. *SE Europe HG*: hunter–gatherer genetic lineages with founders in Near East (a proxy for populations in the northern Subtropical Zone); *W Africa farmer*: genetic lineages for descendent farming populations in West Africa associated mainly with the Bantu expansion; *S Africa HG*: southern African hunter–gatherer lineages. 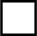 First evidence for farming in West Africa; 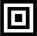 first evidence for farming in northern Subtropical Zone. Redrawn after Gignoux et al. (2011).

The period between 11,000 and 5000 BP was associated with a substantial rise in human population density in the northern Subtropical Zone (as evidenced by both archaeological and genetic data: Kuper & Kröpelin, 2006; Powell et al., 2009; Pereira et al. 2010; Zheng et al. 2012), much of it associated with cattle pastoralism (Manning & Timpson, 2014). Very similar conditions prevailed in the Ganges Plain in northern India (Staubwasser et al., 2003) and in the central regions of China (An et al., 2005). Figure 2 (solid line) plots the population growth over time in the eastern Mediterranean, as determined from coalescence models for mtDNA haplogroups associated with a hunter–gatherer lifestyle: the population began to increase from the beginning of the Humid Period, some 4000 years before the first evidence for substantive agriculture (double square symbol, dated to 7800 years BP; for similar results for North Africa as a whole, see Pereira et al., 2010). In contrast, the hunter–gather populations of sub-Saharan Africa (represented here by San populations from the southern Subtropical Zone: dotted line) remained extremely stable into modern times. Even the farmer lineages in West Africa (dashed line) only began to increase after the first evidence for agriculture in this region around 4000 years ago (single square symbol) (Winchell et al., 2018). In short, it seems that the climatic conditions in the northern Subtropical Zone allowed populations to rise very rapidly, eventually necessitating the development of agriculture to support them (Bocquet-Appel, 2011; Kathayat et al., 2017), whereas within the tropical forest environments nearer the Equator population growth only took off after the introduction of agriculture.

**Figure 3.**
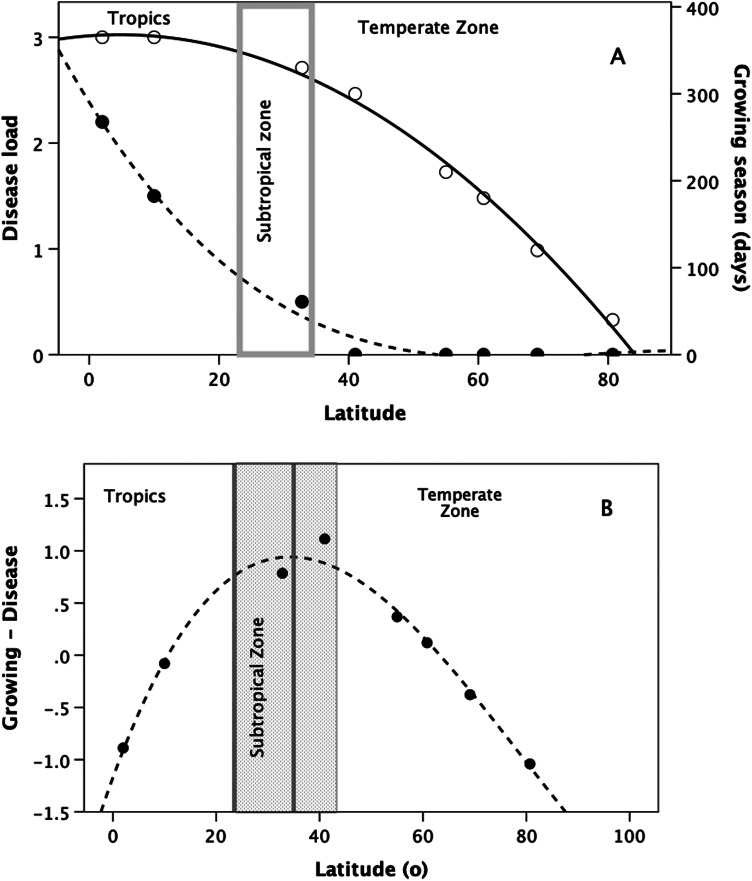
(a) Length of growing season (unfilled symbols, solid line: number of consecutive days when lake water temperature is >9°C) and current disease load (filled symbols, long dashed line: VBPD, summed vector-borne and parasitic diseases) as a function of latitude for various Northern Hemisphere sites. Values are given as standard deviations (standardised to sample mean) so as to allow them to be compared directly. These are well-known geophysical relationships, but I illustrate them here with data from a specific set of sites for disease load (from Bonds et al., 2012), with growing season length matched for the same sites calculated with the equation from from Håkanson and Boulion (2001). Growing season length is largely a function of temperature (and hence seasonality), whereas disease load is largely a function of rainfall (and hence humidity). The grey bar demarcates the Subtropical Zone. (b) Difference between standard deviate (from mean value) for growing season and standard deviate for disease load for each of the sites in (a), plotted against site latitude. The grey lines define the Subtropical Zone; the hatched bar indicates the range of latitudes within which growing season is maximised with respect to disease load. Note that the optimal zone extends from the Tropic of Cancer (latitude 23.5° N) across the Subtropical Zone through to about latitude 42° N: this would include Spain, Sicily, Greece and Turkey.

There is, however, an additional reason why human populations might have grown so rapidly in this region: the latitudinal distributions of disease prevalence and growing season length converge on an optimal balance in the northern Subtropical Zone. Figure 3a illustrates this with contemporary data, with both variables plotted as standard deviates of their respective means so as to allow direct comparison. Both distributions are at their maxima at the Equator. The central Tropics have been historically, and still are, a primary source for new pathogens and vector-borne pandemics (Guernier et al., 2004; Jones et al., 2008) and, historically, were extremely challenging to occupy (Campbell & Tishkoff, 2008). However, while both variables decline with increasing latitude, they do so at very different rates: pathogen load falls rapidly, reaching a minimum at around 50° latitude, whereas growing season remains high well into the subtropical zone, after which it declines steadily from about latitude 30° N towards zero at a latitude of ~85° N.

There is an obvious trade-off here. At very high (i.e. polar) latitudes, the environment is healthy but the climate so adverse that the growing season is measured in days rather than months. Food production rather than disease is the factor that most limits human occupancy; as a result, only hunter–gatherer economies have ever been possible at these latitudes historically. In contrast, at the Equator, the climate is benign and supports year-round agriculture, but populations are subject to heavy disease loads; disease rather than food becomes the principal constraint on population growth. Population growth rates will be optimised where the difference between these two effects is maximised. Figure 3b indicates that the difference is maximised in the Subtropical Zone (the hatched area between the grey bars), extending up to around latitude 40° N (roughly the northern shore of the Mediterranean, including most of modern Turkey and the Levant), creating a unique environment of ecological release. This makes the Subtropical Zone unusually benign for human populations – providing rainfall is high enough to create surplus ground water.

Of course, Figure 3 reflects the current climatic regime, and the climate could have been very different during the early Holocene. In fact, climate models suggest that, during the early Holocene, the climate at low and mid latitudes was wetter, but not necessarily warmer, than it is now (Kuper & Kröpelin, 2006). This largely reflected changes in insolation owing to convergences in the Milankovich cycles. At the height of the African Humid Period around 6000 BP, this was associated with increased seasonality (and hence shorter growing seasons) at high latitudes north of the Equator and cooler temperatures (by up to 2.5°C) in the mid-Tropics (latitude ~10° N; Kitoh & Murakami, 2002). This would have had the effect of giving the growing season graph a more inverted-J shape with its peak in the Subtropical Zone, while at the same time slightly flattening the left-hand side of the disease load graph (Guernier et al., 2004). These differences would not, however, have changed the overall pattern: the northern Subtropical Zone and the area immediately above it would still have been optimal for population growth.

### Origin of phase-1 doctrinal religions

The archaeological evidence suggests that living-group sizes increased dramatically as the Holocene progressed. The earliest settlements were associated with the brief Natufian culture in the Levant (11,000–10,300 BP), with permanent settlements becoming more common and larger as the Holocene progressed. Estimates of settlement size based on site area and number of dwellings suggest that Natufian settlements were broadly within the size range of modern hunter–gatherer living-groups (18–70, Kruijt, 2000; 75–100, Goris-Morris & Belfer-Cohen, 2011) and were associated with a largely foraging-based economy. Once more permanent settlements had been established, there was a rapid increase in community size to ~330 during the PPNA (Pre-Pottery Neolithic phase A, 10,300–9300 BP), rising to ~760 in the early PPNB (9300–8500 BP), and escalating to settlements of 3000–4000 by 8300–7500 BP (Goris-Morris & Belfer-Cohen 2011).

This would have required novel mechanisms for mitigating the stresses experienced if these population concentrations were to persist. While a variety of social institutions (including men's clubs, charismatic leaders, marital obligations and feasting) would undoubtedly have played a role in the early stages, as they do in contemporary village-scale horticultural societies (Dunbar 2022b), doctrinal religions seem to offer a particularly effective mechanism for bonding very large social groups, especially once these exceed the size at which direct personal social contact is no longer possible (~500 individuals: Sutcliffe et al., 2012). Religion is especially effective at bonding very large numbers of anonymous individuals both because formalised rituals up-regulate the endorphin system without requiring physical contact (Charles et al. 2020, 2021) and because the origin stories and other cultural devices they involve exploit the ‘Seven Pillars of Friendship’ (Dunbar 2018) mechanism to create a sense of communality (Dunbar 2022a). That religion may help to bolster communal cohesion and co-action is suggested by evidence that regular attendance at religious services elevates the sense of community membership as well as trust in the wider community, and creates the feeling of having a much wider circle of supportive friends (Dunbar, 2020a).

It should be no surprise, therefore, that we begin to find archaeological evidence in the Levant for temple-like structures from around 9300 BP. These are associated with ritualised burials and cult objects and statues that differ across sites, suggesting highly localised, more doctrinal-type religions (Garfinkel, 1994; Hodder, 2010; Dietrich et al., 2012). This is reflected in the archaeological patterns for trading between Levantine settlements which suggests that, despite a wide trading network, there was a steady decline in the similarity of material cultural between sites (as indexed by the presence of the same types of artefacts) as the Holocene progressed (Coward & Dunbar, 2014: fig. 17.1a). This suggests that settlements became increasingly inward-looking and, trade notwithstanding, more culturally isolated from each other as their size increased over time.

A parallel pattern has been described for coastal Ecuador and Peru in the New World. Prior to ~6.0 ka, there is no evidence for an El Niño weather pattern, indicating a less seasonal, more stable climate typically associated with high rainfall. Populations boomed. After that date, however, ENSO (El Niño southern oscillation) events become a regular feature, creating a more erratic climate (Sandweiss et al., 2001), leading to increasing internal stresses for communities and greater inter-community competition. This period coincides with the foundation of a number of small to medium-sized empires (Sandweiss et al., 2001) associated with evidence for religious sites. The earliest of these temple sites dates from this period (c. 5.8 ka). This sequence has been particularly well documented on the Chiripa peninsula in Lake Titicaca (Bolivia) (Bandy, 2004; see also Chávez, 1988). During the fourth millennium BP, mean village size was around 120, but around 3000 BP there was a rapid increase to about 275, with as many as a quarter of the villages now containing more than 400 people. Within a matter of centuries, a new religious complex, the Yaya–Mama cultural tradition, had made an appearance. This included decorated serving bowls, ceramic trumpets, incense burners and a distinctive style of stone sculpture, as well as a novel form of ceremonial public space (plastered sunken ball courts) associated with temple/storage complexes. It seems that, at this point, organised religion became part of the social toolkit to allow larger communities to be stable enough to survive. As in the Levant, this can be interpreted as a defence against rising levels of inter-community conflict.

### Latitude and religious diversity

Fincher and Thornhill (2008, 2012) have shown that there are striking latitudinal patterns in the distribution of religions, with the number of religions per unit population being positively correlated with pathogen load. They argued that when pathogen levels are high (as they are at the Equator), the best way to reduce exposure is to avoid social exchange (and especially sexual relationships) with communities that have different pathogens. The repeated experience in the last half millennium of Native American populations exposed to ‘mild’ European diseases is testament enough to the costs involved (Alchon, 2003). Using religion as a social isolating mechanism is an extremely effective solution. As pathogen loads decline outside the Tropics, this becomes less necessary, and larger social communities can emerge as a result, leading to fewer religions.

**Figure 4.**
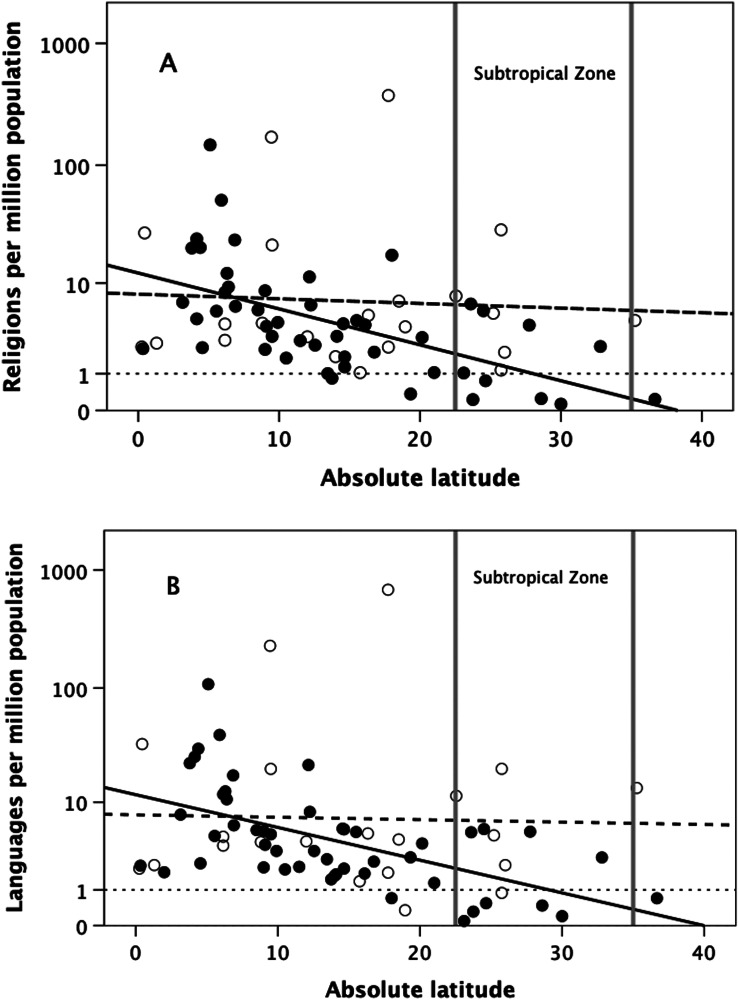
(a) Number of religions per million population and (b) number of languages per million population for individual countries plotted against the latitude of the country's capital city. Filled symbols: Northern Hemisphere countries; unfilled symbols: Southern Hemisphere countries. Solid line is the regression for the Northern Hemisphere countries only; long dashed line is the regression for Southern Hemisphere countries. Regression analyses: (a) log_10_-religions/million: Northern Hemisphere, r^2^ = 0.412 F_1,47_ = 32.9, p<0.0001; Southern Hemisphere: *r*^2^ = 0.004, *F*_1,20_ = 0.001, *p* = 0.778; (b) log_10_-languages/million: Northern Hemisphere, *r*^2^ = 0.367; *F*_1,47_ = 27.3, *p* < 0.0001; Southern Hemisphere, *r*^2^ = 0.002; *F*_1,20_ = 0.035, *p* = 0.854. The vertical grey lines demarcate the Subtropical Zones. Sources: religions, Fincher and Thornhill (2008); languages, Nettle (1999).

Fincher and Thornhill failed to notice two key points about these data that are particularly relevant in the present context. One is that countries north and south of the Equator behave *very* differently. Figure 4a replots the Fincher–Thornhill data as log_10_ number of religions per million population against absolute latitude, with countries that lie north and south of the Equator shown separately. The regression for Northern Hemisphere countries is significantly negative (*r*^2^ = 0.412); that for Southern Hemisphere countries is not (*r*^2^ = 0.004) (for regression statistics, see the figure legend). Notice that the pattern is exactly the same for the density of languages (Figure 4b), and hence inversely for the size of language communities. Since language communities are essentially tribes (a tribe is a linguistic unit), tribes are in effect smaller and more differentiated near the Equator than at higher latitudes.

It seems that it is only in the Northern Hemisphere that the Fincher–Thornhill explanation actually applies. Given that ~50% of the southern zone consists of uninhabitable desert (the high-altitude Andes and their eastern rain shadow, the Karoo desert in southern Africa and the Australian Great Victorian desert) with much of the rest being agriculturally poor quality scrub or veldt-type habitats only suited to cattle pastoralism, it is perhaps not too surprising that doctrinal religions are not found south of the Equator. In fact, almost all of the indigenous tribes that historically (i.e. prior to the fifteenth century) occupied the Southern Subtropical Zone in all three southern continents were (and mostly still are) hunter–gatherers who never adopted a settled village lifestyle.

The second point is that the number of religions (Figure 4a) and the number of languages (Figure 4b) in the Northern Hemisphere both asymptote at around one per million population. Across the whole dataset, the number of religions/million is highly correlated with the number of languages/million (*r* = 0.871, *N* = 72, *p* << 0.0001). Since the regression slope approximates *b* = 1 and the number of languages is a reliable proxy for the number of tribes, the monotonic relationship between the two variables implies that each tribe has its own unique religion. That the number of religions and languages per million population approaches one (the horizontal dotted line) in the Northern Hemisphere seems to dovetail neatly with the finding that large-scale Axial Age religions only emerge when states reach a size of around a million members (Baumard et al., 2015; Whitehouse et al., 2023).

### Towards an explanation for the Axial Age religions

The upward trajectory in settlement size first identified in the early Holocene (see above) continued through the succeeding millennia. By the start of the Bronze Age at 3300 BP, cities like Memphis (in Lower Egypt) and Ebla (in Syria) numbered 30,000–40,000 inhabitants. This period is associated with the rise of the Bronze Age civilisations of the Middle East (the Eblaite, Akkadian, Early Dynastic and Old Kingdom Egypt, and Minoan empires), India (the Harappan Indus Valley civilisation) and China (the pre-Xia empires of the Yellow and Yangste River region) (Inoue et al., 2015).

These developments were, however, brought to an abrupt end by the 4.2 ka Climatic Event that involved a dramatic decline in rainfall around 4200 BP and affected this entire latitudinal belt across Africa (Weiss et al., 1993; Cullen et al., 2000; Lüning & Vahrenholt, 2019) and southern Asia as far east as China (Staubwasser et al., 2003; An et al., 2005; Hong et al., 2000; Malik, 2020). In the Sahara and the Levant, the resulting dry period lasted 300 years and was associated with a progressive lowering of the water table and rapidly reducing lake volumes (Kaniewski et al., 2018), leading eventually to the desertification now observed across the entire region (Riehl et al., 2011; Revel et al., 2014; Lüning & Vahrenholt, 2019).

This period of environmental stress culminated in a centuries-long famine triggered by a rise in temperature and decline in rainfall that set in around 3200 BP, resulting in large-scale population movements and widespread political instability that lasted for some three centuries (Kaniewski et al., 2015). In the eastern Mediterranean, this was associated with the appearance of the ‘Sea People’ whose successive sea-born invasions from the European mainland resulted in the destruction of many coastal cities, significant population displacement and very considerable social and political disruption (Oren, 2000; Kaniewski et al., 2013, 2018; Emanuel, 2014). Similar climate-driven population and economic collapse associated with socio-political upheaval during the same time period led to the demise of both the Harappan civilisation in India (opening the way for subsequent invasions from the northwest by Indo-European pastoralists; Staubwasser et al., 2003) and the rain-fed Qijia agricultural culture in central China (An et al., 2005). A similar pattern is observed in South America: shifts in the weather patterns led to an ENSO regime after ~5000 ka that made it increasingly difficult to produce food in sufficient quantities to support the large urban concentrations that had developed. By 2.8 ka most of the nascent empires that had grown up in the earlier more benign times had collapsed (Burger, 1992).

It may be no accident, then, that this upheaval and stress was associated with the appearance of the Axial Age religions with their greater capacity to enforce good behaviour and create a sense of communality in very large communities (Baumard et al., 2015; Whitehouse et al., 2023).

## Discussion

I have suggested that the latitudinal origins of doctrinal religions in the early Holocene might lie in a unique convergence in the conditions for population growth in the northern Subtropical Zone at this time, and that this was a consequence of the way pathogen load and growing season length are determined by latitude. It is well known that the Tropics are a hothouse for pathogen evolution (Guernier et al., 2004; Jones et al., 2008), owing mainly to its hot climate, high humidity and lack of seasonality. In contrast, the increasingly long, cold winters outside the Tropics put a break on most pathogens’ ability to replicate. Fincher and Thornhill (2008, 2012; Fincher et al., 2008) argued that pathogens place a heavy selection pressure on tropical human populations and anything that will reduce pathogen load will be strongly favoured (see also Campbell & Tishkoff, 2008). Reducing the pool of people from whom one might contract pathogens is largely a matter of reducing the number of individuals with whom one interacts.

Differences in religion and language provide a powerful ideological basis for reinforcing such divisions in locations where the environment is rich enough to support a substantial population. In support of this, Fincher et al. (2008) found that the ratio of individualism vs. collectivism in social attitudes declines with declining disease load, suggesting that people are more inward-looking and community-focussed and less individualistic when disease load is high. In effect, this limits religions within the Tropics to small-scale, tribally based sects and effectively prevents the rise of very large-scale religions of the Axial Age kind (i.e. those associated with Moralising High Gods), although the shift towards a supreme deity in addition to ancestor worship in the Bantu might (given the essential similarity in all Bantu religions) signal a late move in this direction after the adoption of agriculture. Interestingly (although Thornhill and Fincher did not notice it), this only seems to happen in the Northern Hemisphere. In the Southern Hemisphere, environments are ecologically too poor to support more than a low-density, hunter–gatherer economy prior to the Bantu and European cattle-based invasions between the sixteenth and eighteenth centuries AD. As a result, populations there never achieved the densities at which raiding becomes a sufficiently intrusive problem to favour concentration in settlements, and hence the need for doctrinal religions. Similarly, centralised empires of a size sufficient to trigger the rise of phase-2 doctrinal religions did not arise in sub-Saharan Africa until well into historical times following the adoption of agriculture, by which time existing Axial Age religions (notably Islam and, later, Christianity) were already available.

Historically, high population growth rates have resulted in increased levels of inter-community conflict as burgeoning populations seek new land to occupy, usually at the expense of existing inhabitants. The archaeological record is replete with evidence for conflict between indigenous hunter–gatherer populations and agricultural invaders during the middle Neolithic in both Europe (Teschler-Nicola et al., 1999; Wahl & Trautmann, 2012; Fry & Söderberg, 2013; Meyer et al., 2015; Alt et al., 2020) and (somewhat later) North America (Milner et al., 1991; Melbye & Fairgrieve, 1994). In most cases, the response of the indigenous population to these kinds of external threat has been to retreat into more defensible positions (Keeley, 1996; Johnson & Earle, 2001). Arab and African slaving raids in West and Central Africa during the eighteenth and nineteenth centuries, for example, resulted in many tribes retreating into more defendable mountainous locations (de Aguilar, 1994; MacEachern, 2011; Wade, 2019). The Zuni responded similarly by retreating into a mountain fastness (Corn Mountain) during the Pueblo Revolt against the Spanish in New Mexico in the 1680s (Liebmann et al., 2005). In other cases, populations gathered into ever larger defended villages: examples include the many walled cities in the Levant and the hill forts of Iron Age Europe (Keeley, 1996; Johnson & Earle, 2001; Müller, 2016). Although the claim advanced here does not depend on this particular explanation (it requires only that people gather in permanent settlements of increasing size and that there be some reason why they should need to do so), the archaeological and historical evidence for conflict as a driver provides strong *bona fide* support for the claim by providing a reason why aggregations should have become necessary.

Doctrinal religions might play a role in alleviating the stresses of group living in either of two distinct ways. One is by providing a form of top-down discipline in the form of a god who encourages good behaviour through fear of divine punishment (Johnson, 2005; Johnson & Bering, 2009). Although this form of top-down enforcement probably emerged in its most effective form only with the later (phase-2) Axial Age religions, even a phase-1 god who simply demands regular sacrifices probably helps to impose discipline and adherence to the local mores – not least because they create a sense of belonging to a community through communal rituals. The other mechanism derives from the fact that the rituals of religion directly influence social bonding as a form of bottom-up commitment via their effect on the brain's endorphin system. Up-regulation of the endorphin system creates a personalised sense of obligation that encourages community members to adhere to the locally agreed mores (Charles et al., 2020, 2021; Dunbar, 2020a, 2022a; Baranowski-Pinto et al., 2022). This second mechanism may have been more important during the early doctrinal phase because the escalating stresses that settlements above the size of hunter–gatherer bands face may be more effectively defused by bottom-up personal commitment to the community than by enforced top-down policing provided by a Moralising High God (Dunbar, 2022a). Top-down policing by an elite invariably produces resentment and resistance, and is difficult to implement in the absence of what amounts to an organised police force.

That religion might be a direct response to external threats as a mechanism for increasing community cohesion and coaction has been suggested by a number of studies. In three contemporary societies, Henrich et al. (2019) found that religiousness (religiosity) was greater among individuals who had been directly exposed to a local civil war than among individuals who had not had direct experience of the conflict. Similarly, Jackson et al. (2021) found that external threats increased social conformity and its public enforcement in both historical and contemporary societies, and this in turn preceded, and was a strong predictor of, future strength of belief in a punitive God. Similarly, in a large-scale study of more than 190 communities drawn from 97 countries, Neuberg et al. (2014) found that the degree of ‘religious infusion’ (the extent to which religion permeates private and public life, for example by justifying prejudice and discrimination) correlates significantly with both the extent to which the society's worldview clashes with those of its neighbours and the extent to which it is in competition with its neighbours for resources and power.

## Data Availability

The data are provided in the online Supplementary Information file *SI Datafile.*

## Supplementary material

For supplementary material accompanying this paper visit https://doi.org/10.1017/ehs.2023.13. click here to view supplementary material

## Research transparency and reproducibility

All data, materials and data are available in the Supplementary Information file *Datafile.*
